# The Scientific American Reference Book

**Published:** 1895-03

**Authors:** 


The Scientific American Reference Book. Price, 25 cents.

This is a most useful little bound book of 150 pages, comprising, probably, the most extensive variety of standard, practical, condensed information ever furnished to the public for so small a price. The following is an abbreviated list of contents:

1. The Last Census of the United States (1890); 2. Table of Occupations; 3. Table of Cities having over 10,000 inhabitants; 4. Map of the United States. Miniature outline; 5. The United States Patent Laws (full text); 6. The Ornamental Design Patent Law (full text); 7. The United States Trade-mark Law (full text); 8. The Label Copyright Law (full text); 9. The General Copyright Law of the United States (full text); 10. The Principal Mechanical Movements; 11. The Steam Engine; 12. Geometry; 13. Horse-Power; 14. Knots; 15. Tables of Weights and Measures; 16. Valuable Tables; 17. Medallion Portraits of Distinguished American Inventors; 18. Engravings of Capitol, Washington; 19. Miscellaneous Information.

*The Scientific American Reference Book*, price only 25 cents, may be had of News Agents in all parts of the country, and of the undersigned. Sent by mail on receipt of the price. Address Munn & Co., 361 Broadway, New York.

